# Dimerization of the transmembrane domain of amyloid precursor protein is determined by residues around the γ-secretase cleavage sites

**DOI:** 10.1074/jbc.M117.789669

**Published:** 2017-08-08

**Authors:** Yan Yan, Ting-Hai Xu, Kaleeckal G. Harikumar, Laurence J. Miller, Karsten Melcher, H. Eric Xu

**Affiliations:** From the ‡ Key Laboratory of Receptor Research, VARI-SIMM Center, Center for Structure and Function of Drug Targets, Shanghai Institute of Materia Medica, Chinese Academy of Sciences, Shanghai 201203, China,; the § University of Chinese Academy of Sciences, 19A Yuquan Road, Beijing 100049, China,; the ¶ Center for Cancer and Cell Biology, Innovation and Integration Program, Van Andel Research Institute, Grand Rapids, Michigan 49503, and; the ‖ Department of Molecular Pharmacology and Experimental Therapeutics, Mayo Clinic, Scottsdale, Arizona 85259

**Keywords:** Alzheimer disease, amyloid precursor protein (APP), amyloid-beta (AB), dimerization, transmembrane domain, C99, FAD-linked mutations, epsilon-cleavage assay

## Abstract

One of the hallmarks of Alzheimer's disease is the formation of extracellular amyloid plaques that consist mainly of abnormally aggregated forms of amyloid β (Aβ) peptides. These peptides are generated by γ-secretase–catalyzed cleavage of a dimeric membrane-bound C-terminal fragment (C99) of the amyloid precursor protein. Although C99 homodimerization has been linked to Aβ production and changes in the aggregation-determining Aβ42/Aβ40 ratio, the motif through which C99 dimerizes has remained controversial. Here, we have used two independent assays to gain insight into C99 homodimerization in the context of the membrane of live cells: bioluminescence resonance energy transfer and Tango membrane protein–protein interaction assays, which were further confirmed by traditional pull-down assays. Our results indicate a four-amino acid region within the C99 transmembrane helix (^43^TVIV^46^) as well as its local secondary structure as critical determinants for homodimerization. These four amino acids are also a hot spot of familial Alzheimer's disease–linked mutations that both decrease C99 homodimerization and γ-secretase cleavage and alter the initial cleavage site to increase the Aβ42/40 ratio.

## Introduction

One of the hallmarks of Alzheimer's disease (AD)^4^ is the formation of extracellular amyloid plaques that mainly consist of the aggregated forms of amyloid β (Aβ) peptides (1, 2). These peptides are proteolytic products of the amyloid precursor protein (APP), a single-pass transmembrane protein with a large N-terminal extracellular domain (ECD) and a small intracellular region. APP is successively cleaved by proteases called secretases, and both full-length APP and its diverse cleavage products have distinct biological functions with incompletely understood roles in neuronal homeostasis (3). APP cleavage can follow two pathways referred to as the non-amyloidogenic and the amyloidogenic pathways. In the non-amyloidogenic processing pathway, APP is first cleaved in its extracellular domain by α-secretase to generate a long-secreted form of APP (sAPPα) and an 83-amino acid membrane-bound C-terminal fragment that is subsequently cleaved by γ-secretase within the membrane to generate the extracellular p3 peptide and the 50-amino acid APP intracellular domain (AICD). In the alternative amyloidogenic pathway, APP cleavage is initiated by β-secretase, generating sAPPβ and a 99-amino acid C-terminal fragment (C99) that is further cleaved sequentially by γ-secretase to generate AICD and 37–42-amino acid-long extracellular Aβ peptides (4–6).

Generation of Aβ peptides and Aβ plaques is believed to have a causal role in the development of AD, as (i) duplications of the *APP* gene locus (7, 8), (ii) mutations in the C99-encoding region of APP, and (iii) mutations in the C99-cleaving γ-secretase (9, 10) can cause familial AD (FAD). The mutations can be examined in detail at the Alzforum database online (http://www.alzforum.org/mutations) (46).^5^

Moreover, FAD mutations in the C99 region consistently skew cleavage to increase the ratio of the highly fibrillogenic and aggregation-prone Aβ42 relative to the main Aβ isoform, Aβ40, indicating that Aβ aggregation is a critical factor in AD pathogenesis. APP has three dimerization domains in its ECD (11–13). In addition, APP also dimerizes through the C99 region, and membrane-bound C99 forms stable homodimers in the absence of the ECD. Importantly, C99 dimerization has been linked to Aβ production and changes in the Aβ42/Aβ40 ratio (13–15).

Apart from C99, γ-secretase cleaves >90 other substrate proteins (16). These proteins have little if any sequence similarity, yet their most prominent members (C99, Notch, ErbB4, and others) have been demonstrated to all homodimerize through their transmembrane domains (TMDs) (17–21). In the case of C99, TMD-mediated homodimerization motifs have been investigated by nuclear magnetic resonance spectroscopy of isolated peptides from the C99 TMD (22–24), by molecular modeling (17, 25), and by biochemical interaction assays, yet the dimerization motif has remained elusive. C99 has three G*XXX*G motifs, which have been implicated as general TMD dimerization motifs. However, mutational analyses indicate that these motifs do not influence C99 dimerization (13, 26), suggesting that other motifs or residues are crucial for homodimerization in the membrane environment.

In this study, we used three independent assays to gain insight into C99 homodimerization: two assays in the context of the membrane of live cells, bioluminescence resonance energy transfer (BRET) and the sensitive Tango membrane protein-protein interaction assay (27), as well as a standard *in vitro* pull-down assay. Our results indicate local secondary structure and a four-amino acid region in the TMD, ^43^TVIV^46^ (TVIV), which is a hot spot of FAD-linked mutations (28), as critical determinants for C99 homodimerization.

## Results

### C99 dimerization can be analyzed in the membrane of live cells by Tango and BRET assays

**Figure 1.**
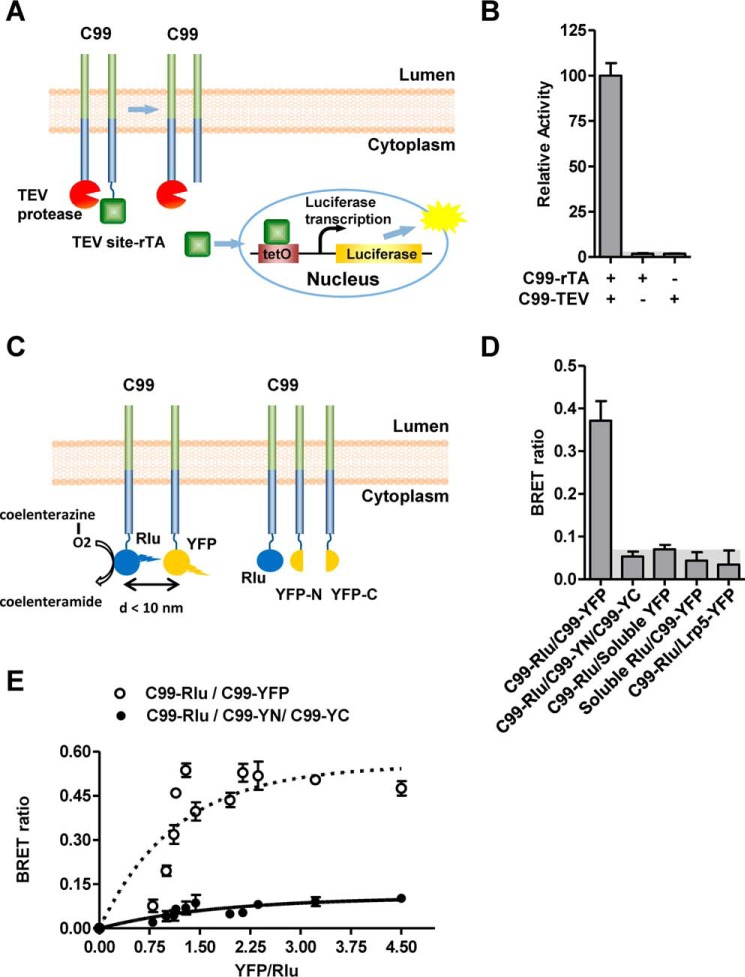
**Validation of C99 homodimerization.** *A*, schematic of the Tango interaction assay. Upon membrane cleavage of the C99 hybrid protein by TEV protease, the rTA transactivator protein is released from the membrane into the cytoplasm. This allows rTA to enter the nucleus and bind the *tetO* DNA-binding site upstream of an integrated luciferase reporter gene to stimulate luciferase reporter gene activity as measured by luminescence. *B*, validation of C99 dimerization by Tango assay. *C*, schematic of the BRET assay. *Left*, two-hybrid BRET assay that detects both homodimerization and oligomerization; *right*, three-hybrid BRET that requires oligomerization for signal generation. C99–Rlu and C99–YFP were coexpressed, and luminescence signals were measured at 480 nm (Rlu emission) and 535 nm (YFP emission) upon addition of the Rlu substrate coelenterazine. Controls are non-associating proteins (soluble fluorescent proteins and the unrelated, membrane-bound Lrp5–YFP fusion protein). *D*, the BRET ratio indicates strong C99 dimerization, which was confirmed by saturation BRET analysis (*E*). *Error bars*, S.E. (*n* = 6). *Shaded areas*, background signals.

Because analysis of C99 dimerization in reconstituted systems, extracts, and *in silico* (18, 19, 23, 24, 29–32) has not been able to identify the determinant(s) of dimerization, we wanted to analyze C99–C99 interactions in living cells. We first adopted a Tango protein–protein interaction assay (27) to determine C99 dimerization. In this assay, we fused the C terminus of C99 to either tobacco etch virus (TEV) protease (C99–TEV) or to a TEV protease cleavage site (TEV site) followed by the synthetic transcriptional activator, reverse tetracycline-controlled transactivator (rTA) (C99–TEV site–rTA; see Fig. 1*A*). C99 dimerization positions TEV protease of one monomer next to the TEV site of the other monomer to allow its efficient cleavage and therefore release of rTA. Membrane-released rTA enters the nucleus and binds and activates a luciferase reporter gene, ultimately converting the dimerization event in the membrane into a quantifiable luminescence signal (Fig. 1*A*). Whereas expression of only C99–TEV site–rTA or C99–TEV alone generated very low background luminescence levels, co-expression resulted in a dramatically increased signal, consistent with strong C99 dimerization (Fig. 1*B*), suggesting C99–C99 interaction in live cells.

To validate this result by another assay in the context of the intact membrane, we fused the C terminus of C99 to either *Renilla* luciferase (Rlu) or yellow fluorescent protein (YFP). When Rlu and YFP are brought into close proximity by C99 dimerization, Rlu bioluminescence mediates an YFP BRET signal, resulting in increased YFP and decreased Rlu light emission (expression-independent BRET ratio; Fig. 1*C*). Consistent with the data in Fig. 1*B*, coexpression of C99-Rlu and C99-YFP caused a strongly increased BRET ratio (Fig. 1*D*). To further test whether C99 can also form oligomers, we used a three-hybrid BRET assay, in which we coexpressed C99-Rlu with C99 fused to either the N-terminal (YFP-N) or C-terminal half (YFP-C) of YFP. Only when all three hybrid proteins are in close proximity can YFP-N and YFP-C reconstitute YFP and allow it to function as a BRET acceptor for adjacent Rlu (see Fig. 1*C*). As seen in Fig. 1*D*, coexpression of C99-Rlu, C99-YFP-N, and C99-YFP-C did not increase the BRET ratio above background, indicating that C99 dimerizes but does not oligomerize. To further distinguish between true interactions and random collisions between hybrid proteins, we performed BRET saturation assays (Fig. 1*E*). Upon titration of a constant amount of Rlu with increasing amounts of YFP, random collisions cause non-saturating, quasi-linear signal increases, whereas true interactions generate plateau-reaching, hyperbolic signals. The two-hybrid, but not the three-hybrid, interaction rapidly reached a plateau, confirming that it is indeed due to specific C99–C99 interaction (Fig. 1*E*).

### The N and C termini of C99 are not required for dimerization

**Figure 2.**
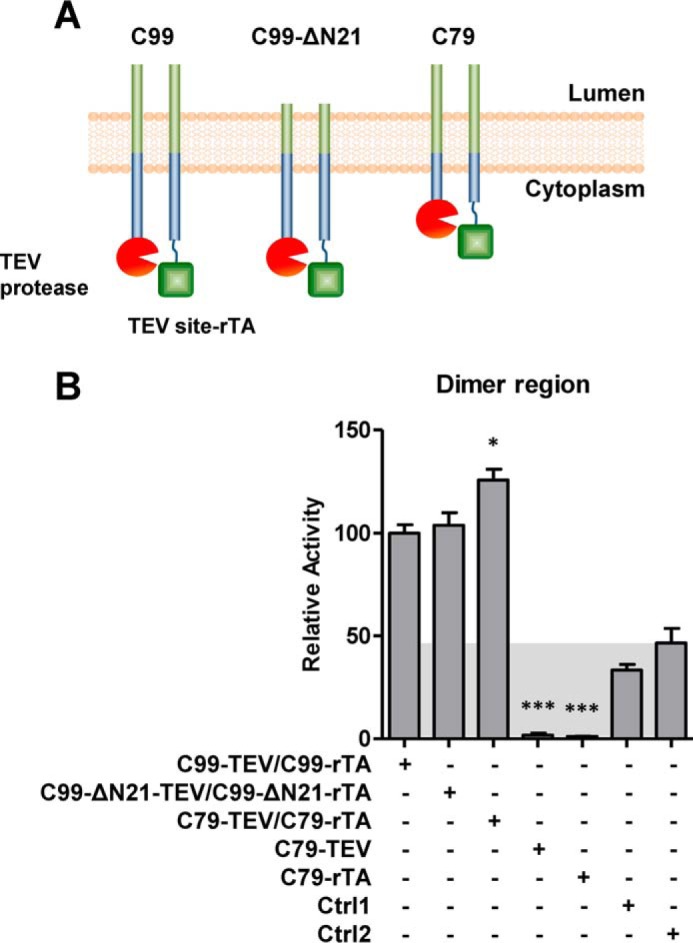
**The transmembrane domain mediates C99 homodimerization.** *A*, schematic illustration of Tango constructs with N- and C-terminally truncated C99. *B*, Tango dimerization assay of full-length and truncated C99. *Ctrl1*, nonspecific interaction between C99–TEV site–rTA and human visual arrestin fused to TEV protease. *Ctrl2*, nonspecific interaction between C99–TEV and human rhodopsin–TEV site–rTA. *Error bars*, S.E. (*n* = 3). *, *p* < 0.05; ***, *p* < 0.001 (two-tailed Student's *t* test *versus* WT). *Shaded areas*, background signals.

To define the dimer interface, we first deleted either the N-terminal 21 amino acids (C99-ΔN21) or the C-terminal 20 amino acids (C79) from the Tango assay constructs (Fig. 2*A*). Both truncated proteins efficiently dimerized, and C79 even generated an increased interaction signal, consistent with a previous report that C-terminal truncation increases APP dimerization (13). Together, these data indicated that the TMD with immediately adjacent residues is sufficient for dimerization. We noted that this fragment contains the minimum C99 region that is required and sufficient for γ-secretase cleavage (Glu^22^–Lys^55^) (33), consistent with the proposed role of C99 homodimerization in binding and cleavage by γ-secretase. We therefore focused our further analysis on this region.

### Secondary structure requirements for C99 homodimerization determined by proline scanning mutagenesis

**Figure 3.**
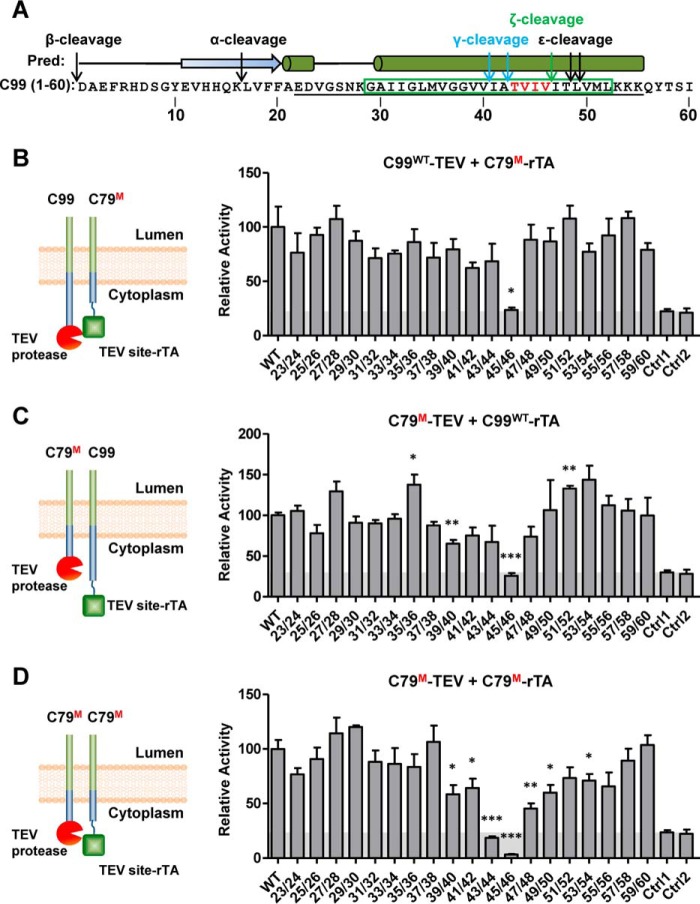
**Identification of local secondary structure requirements for C99 homodimerization.** *A*, sequence of residues 1–60 of C99 with predicted secondary structure elements and α-, β-, and γ-secretases cleavage sites. Loops, α-helices, and β-strands are indicated as *lines*, *cylinders*, and *arrows above* the sequence. Key residues are shown in *red. B*, interaction between WT C99–TEV and double-proline-mutated (*M*) C79–TEV site–rTA. *C*, interaction between double-proline-mutated (*M*) C79–TEV and WT C99–TEV site–rTA. *D*, interaction between double-proline-mutated (*M*) C79–TEV and double-proline-mutated (*M*) C79–TEV site–rTA. In all three combinations, most of the double proline mutant proteins still formed stable dimers, with the exception of the key residues Thr^43^, Val^44^, Ile^45^, and Val^46^. *Ctrl1*, nonspecific interaction between C99–TEV site-rTA and human visual arrestin fused to TEV protease. *Ctrl2*, nonspecific interaction between C99–TEV and human rhodopsin–TEV site–rTA. The *numbers* indicate the proline mutation sites. *Error bars*, S.E. (*n* = 3). *, *p* < 0.05; **, *p* < 0.01; ***, *p* < 0.001 (two-tailed Student's *t* test *versus* WT). *Shaded areas*, background signals.

**Figure 4.**
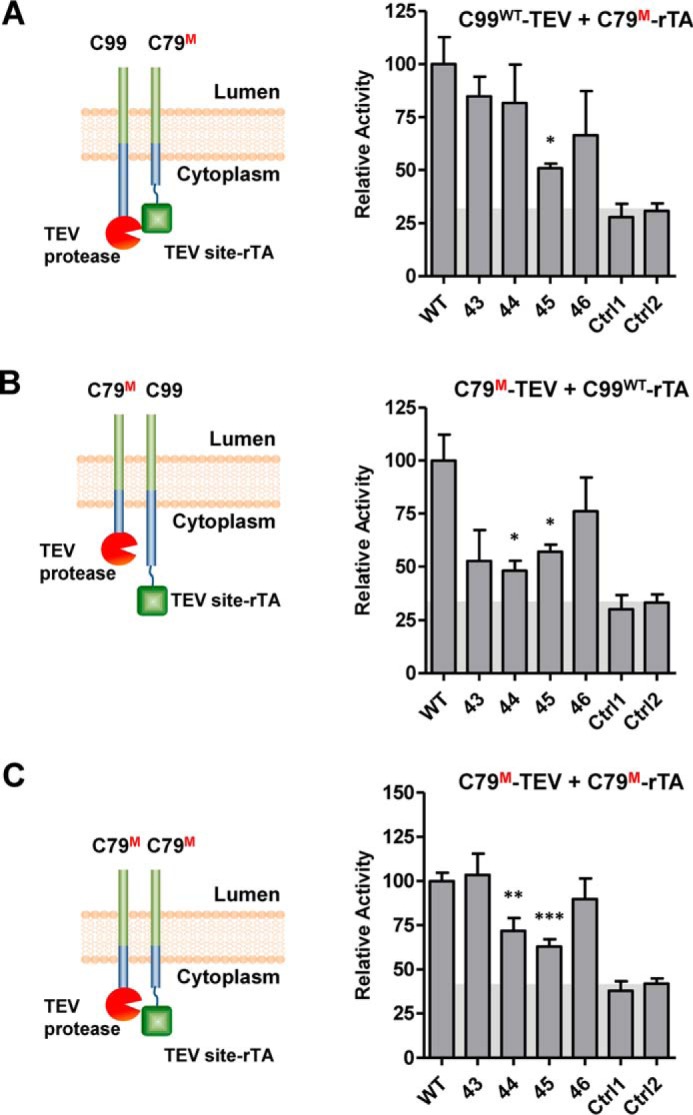
**C99 single proline TVIV mutations cause moderate dimerization defects.** *A*, interaction between WT C99–TEV and mutant (*M*) C79–TEV site-rTA. *B*, interaction between proline-mutated (*M*) C79–TEV and WT C99–TEV site-rTA. *C*, interaction between mutant (*M*) C79–TEV and mutant (*M*) C79–TEV site–rTA. *Ctrl1*, nonspecific interaction between C99–TEV site–rTA and human visual arrestin fused to TEV protease. *Ctrl2*, nonspecific interaction between C99–TEV and human rhodopsin–TEV site–rTA. The *numbers* indicate the proline mutation sites. *Error bars*, S.E. (*n* = 3). *, *p* < 0.05; **, *p* < 0.01; ***, *p* < 0.001 (two-tailed Student's *t* test *versus* WT). *Shaded areas*, background signals.

**Figure 5.**
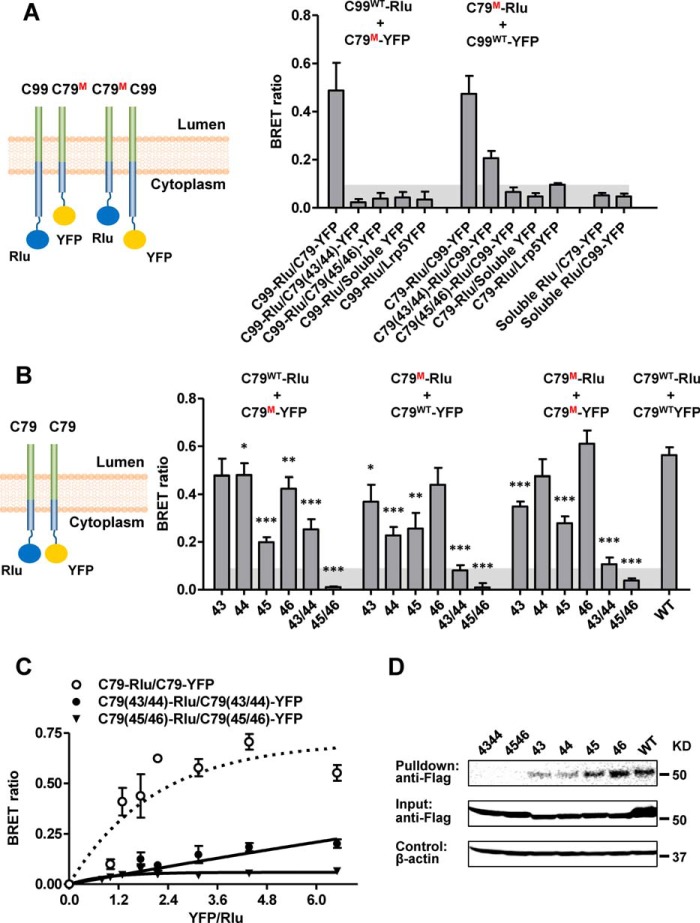
**BRET and pulldown assay validation of C99 proline mutations.** *A*, BRET interaction between WT C99-Rlu and proline-mutated (*M*) C79–YFP or proline-mutated (*M*) C79–Rlu with WT C99–YFP. *B*, BRET interaction between C79–Rlu and C79–YFP with different proline mutations. *C*, saturation BRET control. *D*, pulldown assay. Biotinylated Avi-tagged C99 proteins and associated C99–TEV site–rTA-FLAG were recovered on Streptavidin MagBeads (GenScript) and eluted with SDS sample buffer. Co-purified C99–TEV site–rTA–FLAG proteins were detected by anti-FLAG immunoblotting. β-Actin input levels serve as loading controls. The *numbers* indicate the proline mutation sites. *Error bars*, S.E. (*n* = 6). *, *p* < 0.05; **, *p* < 0.01; ***, *p* < 0.001 (two-tailed Student's *t* test *versus* WT). *Shaded areas*, background signals.

γ-Secretase cleaves >90 different proteins that are all type-I TM proteins but share little sequence similarity (16). Therefore, γ-secretase cleavage does not depend on any extended specific amino acid motif, but more likely on TM domain conformational state or secondary structure (34). We therefore first used double proline scanning mutagenesis by replacing pairs of consecutive residues with prolines to induce kinks that destabilize both α-helical and β-strand secondary structure elements. We introduced these mutations into C79–TEV site–rTA to test dimer formation with wild-type C99–TEV (Fig. 3*B*), into the corresponding region of C79–TEV to test dimer formation with wild-type C99–TEV site–rTA (Fig. 3*C*), and into both C79–TEV site–rTA and C79–TEV (Fig. 3*D*). Whereas most mutations did not significantly change dimerization, mutations in the region from Val^39^ to Val^50^, and especially I45P/V46P and to a lesser degree T43P/V44P, strongly reduced the dimerization signal (Fig. 3, *B–D*). These four amino acids (TVIV; highlighted in Fig. 3*A*) are a hot spot for FAD-linked APP mutations (28) (also see Fig. 10*A*); they are flanked by γ-secretase cleavage sites and are located in a predicted α-helix (Fig. 3*A*). Single proline mutations of these four residues caused more moderate dimerization defects (Fig. 4) yet followed the same trend, with mutation of Ile^45^ having the largest effect. To validate the dimerization defects of the TVIV double proline mutations, we first used BRET assays, which indicated an almost complete C99–C79 interaction signal loss (Fig. 5*A*), as well as single proline mutations, which caused smaller, yet still highly significant C79 dimerization defects, especially for I45P (Fig. 5*B*), in good agreement with our Tango assay data. As expected, I45P/V46P and T43P/V44P mutations in the context of C79-Rlu and C79-YFP failed to show signal saturation and followed quasi-linear signal increases, further confirming that they lost the ability to dimerize (Fig. 5*C*).

We also used a pulldown assay to confirm the dimerization defects of the TVIV double proline mutations in the context of cell lysates. In this experiment, we coexpressed wild-type and mutant C79–TEV site–rTA–FLAG with an Avi biotinylation tag–C99 fusion protein for in-cell Avi tag biotinylation (see “Experimental procedures”). We then recovered biotinylated Avi–C99 with associated non-biotinylated C99–TEV site–rTA–FLAG proteins on Streptavidin MagBeads. After extensive washing, we eluted complexes with SDS and detected associated C99–TEV site–rTA–FLAG by immunoblotting. In excellent agreement with the BRET assay, the single proline mutant proteins, with the exception of V46P, were less efficiently pulled down than wild-type C99, and double proline mutant proteins were not recovered at the sensitivity level of this assay (Fig. 5*D*).

**Figure 6.**
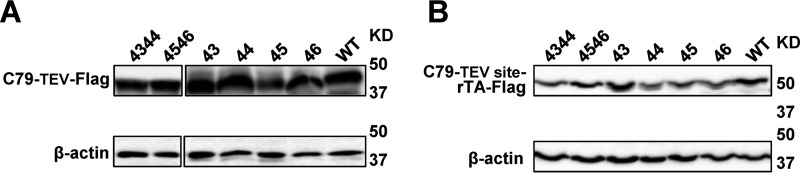
**Expression analysis of C99 WT and C79 mutant proteins.** Shown is an immunoblot of lysates from cells expressing WT (C99) and mutant C79–TEV–FLAG (*A*) or C79–TEV site–rTA–FLAG (*B*), using anti-FLAG antibody for protein expression detection and anti-β-actin antibody for normalization. The *numbers* indicate the proline mutation sites.

**Figure 7.**
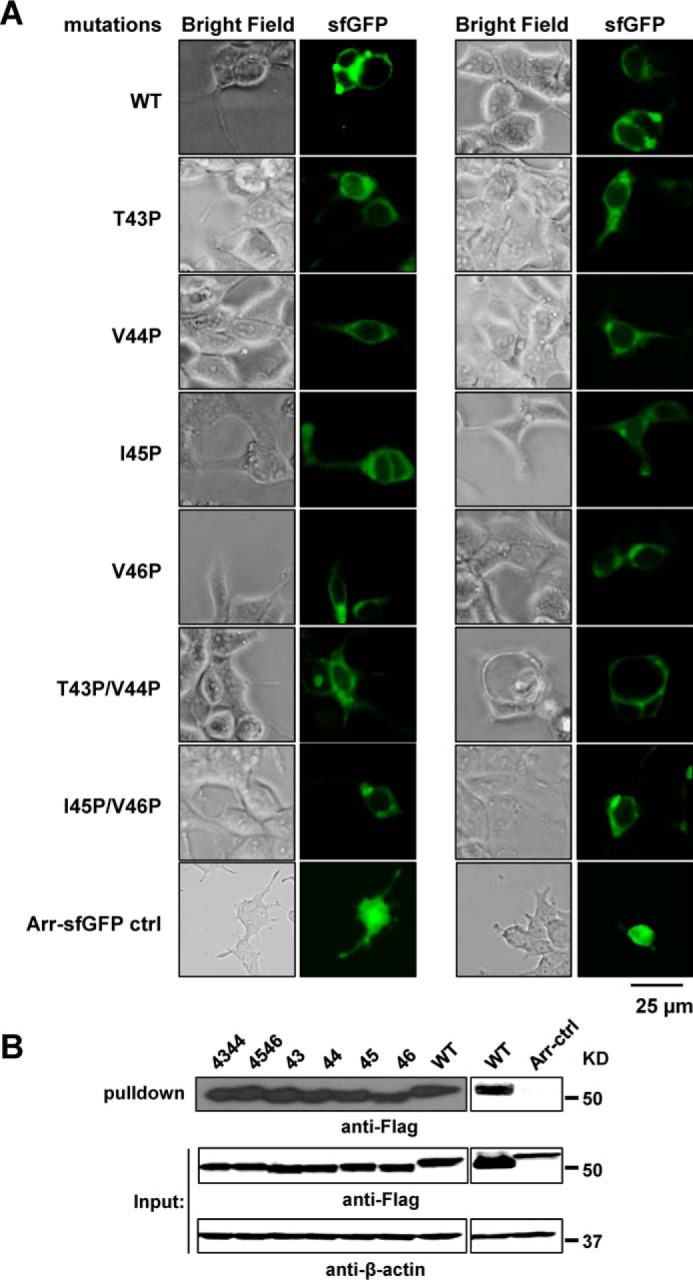
**Membrane expression of WT C99 and mutant C79.** *A*, fluorescence microscopy: C99 proteins were C-terminally fused to sfGFP. For each mutant, the *right panel* shows the GFP channel and the *left panel* shows the same view in the bright field channel 12 h after transfection. C-terminally sfGFP-tagged arrestin (*Arr-sfGFP*) serves as cytoplasmic expression control. *B*, surface biotinylation. Cells expressing C99 fusion proteins were surface-labeled with S–S–linked biotin. After removal of free biotinylation reagent, cells were lysed, and biotinylated proteins were recovered on Streptavidin MagBeads, eluted by reduction of the S–S link in SDS sample buffer, and detected by immunoblotting. FLAG-tagged Arr serves as cytoplasmic expression control. The *numbers* indicate the proline mutation sites.

We determined total levels of wild-type and mutant C99 proteins by immunoblotting (Fig. 6). We further determined the localization of C99 proteins both by fluorescence (Fig. 7*A*) and by surface biotinylation (Fig. 7*B*). Both assays demonstrated that all proteins were membrane-expressed. Whereas the total levels of C99 proteins showed small variations, there was no correlation between relative expression levels and dimerization signals, implying that the observed dimerization defects are not simply due to lack of expression or mislocalization.

### Both TVIV secondary structure and side chains are important for dimerization

**Figure 8.**
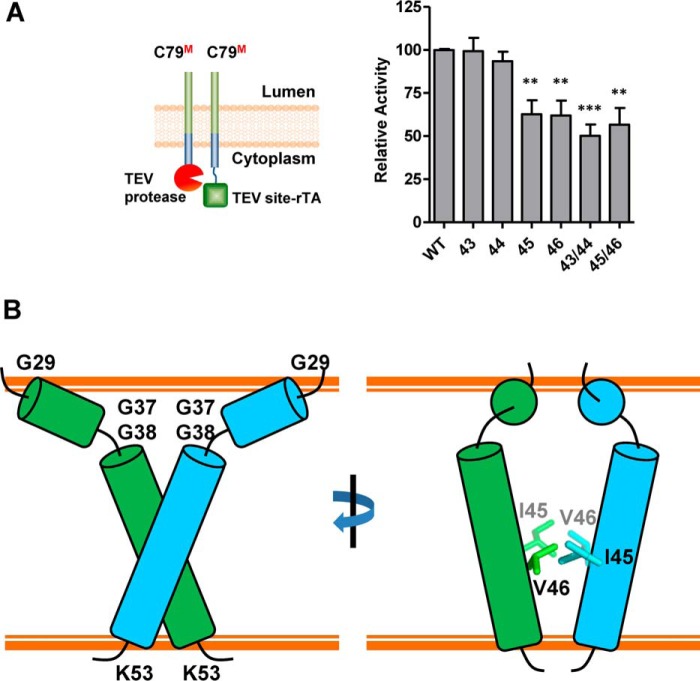
**A schematic model for C99 homodimerization.** *A*, TVIV alanine substitutions in the context of C79 constructs decrease Tango assay dimerization signals. The *numbers* indicated the alanine mutation sites. *Error bars*, S.E. (*n* = 6). **, *p* < 0.01; ***, *p* < 0.001 (two-tailed Student's *t* test *versus* WT). *B*, schematic model based on Phyre2 structure prediction. The transmembrane helices are shown as *green* and *cyan cylinders*, and loop regions, including the GG kink, are presented as *lines*. Key residues are represented as *stick models*, with *light colors* indicating the residue pair in the *back plane*.

To test whether the effect of proline mutations is indeed due to local secondary structure disruption or, alternatively, to changes in amino acid side chains, we introduced single and double alanine mutations into TVIV, as alanine does not disrupt helical structure. Interestingly, single alanine mutations had similar effects as single proline mutations, yet, in striking contrast to double proline mutations, double alanine mutations did not further decrease C79 dimerization (Fig. 8*A*). Collectively, this suggests that both the side chains of the TVIV motif, and especially of Ile^45^, as well as local TVIV secondary structure contribute to dimer formation.

Insight into the dimeric structure of C99 and its proteolytic processing by γ-secretase would probably shed light on Aβ generation and AD pathology. Based on the C99 topology (22) and Phyre2 prediction (35) together with the results above, we propose a model for C99 homodimerization shown in Fig. 8*B*. In the schematic presentation of the Phyre2 model, the transmembrane dimer consists of a T-shaped helix pair, in which the helices cross each other at the ^43^TVIT^46^ interface, with residues Ile^45^ and Val^46^ facing each other.

### C99 homodimerization mutants are compromised γ-secretase substrates

**Figure 9.**
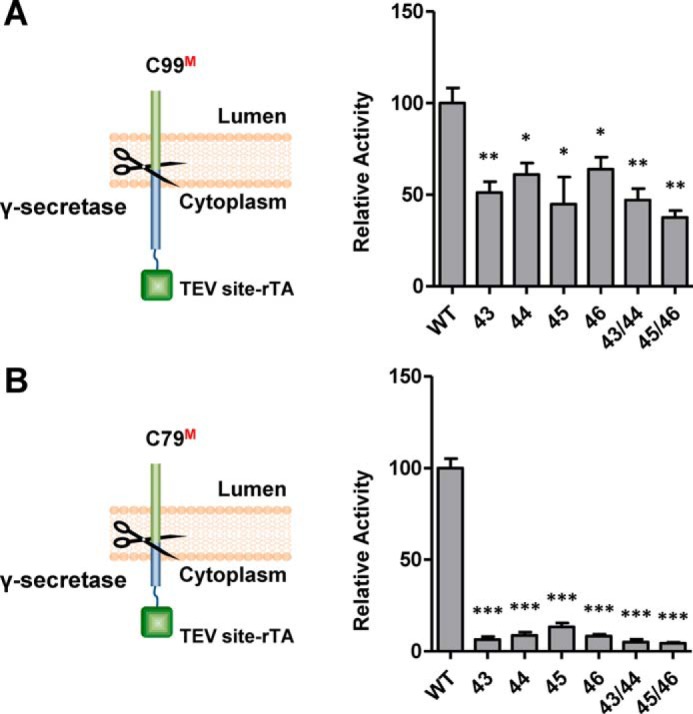
**Alanine and proline substitutions in the TVIV motif reduce cleavage by γ-secretase.** *A*, *left*, schematic illustration of the ϵ-cleavage assay. Cleavage of C99–TEV site–rTA by endogenous γ-secretase releases free AICD–TEV site–rTA, which stimulates the luciferase reporter activity in the nucleus. *Right*, alanine substitutions in the TVIV motif decreased the efficiency of γ-secretase cleavage. The *numbers* indicate the positions of alanine substitution. *B*, proline mutations at the TVIV motif in the context of C79, which is a better γ-secretase substrate than C99, dramatically decreased the efficiency of γ-secretase cleavage. The numbers indicated the proline mutation sites. *Error bars*, S.E., *n* = 6. *, *p* < 0.05; **, *p* < 0.01; ***, *p* < 0.001 (two-tailed Student's *t* test *versus* WT).

**Figure 10.**
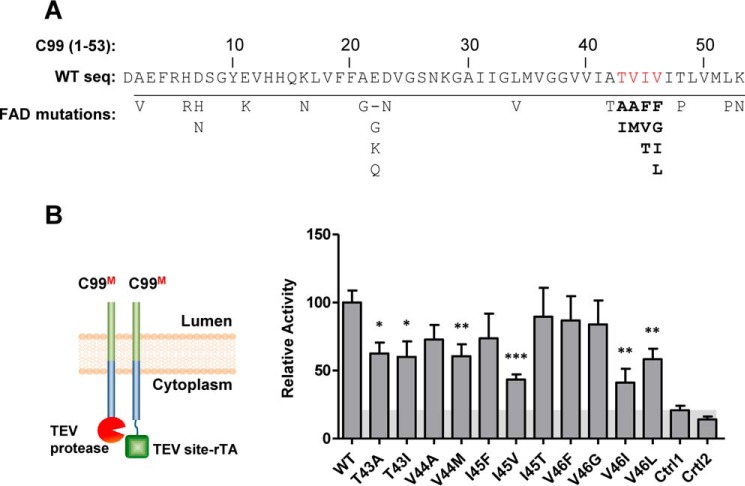
**FAD-linked TVIV mutations reduce C99 homodimerization.** *A*, location of FAD-linked C99 mutations. Shown is a list of FAD-linked APP mutations within the first 53 amino acids of C99 as listed in the Alzforum database (http://www.alzforum.org/mutations).^5^ The TVIV motif residues are *highlighted* in *red*. Note that a large fraction (11 of 28; *boldface type*) of the C99 FAD-linked mutations localize to the TVIV motif. *B*, all FAD-linked mutations in the C99 TVIV dimer motif reduced Tango assay dimerization signals. *Ctrl1*, nonspecific interaction between C99–TEV site–rTA and human visual arrestin fused to TEV protease. *Ctrl2*, nonspecific interaction between C99–TEV and human rhodopsin–TEV site–rTA. *Error bars*, S.E. (*n* = 6). *, *p* < 0.05; **, *p* < 0.01; ***, *p* < 0.001 (two-tailed Student's *t* test *versus* WT).

To test the effect of dimer disruption on γ-secretase cleavage, we tested our wild-type and mutant C99 and C79 Tango constructs in the cell-based ϵ-cleavage assay (28) (Fig. 9). All single and double alanine mutations in the TVIV motif significantly reduced γ-secretase cleavage in the context of C99 (Fig. 9*A*), and all single and double proline mutations almost abrogated cleavage in the context of C79 (Fig. 9*B*), cleavage defects that resemble the severe dimerization defects seen in the BRET assay (compare Figs. 5 and 9). Next, we extended this analysis to the 11 known FAD-linked mutations in residues 43–46 shown in *boldface type* in Fig. 10*A*). As shown in Fig. 10*B*, at least six of these mutations significantly decreased dimerization even in the less sensitive C99–C99 Tango interaction assay. Because all of these mutations decreased C99 cleavage and dramatically skewed the Aβ42/Aβ40 ratio (28), perturbed dimerization in these FAD mutations may change γ-secretase cleavage toward more toxic Aβ processing and thus lead to the AD pathology.

### Notch TM fragments also stably self-associate

**Figure 11.**
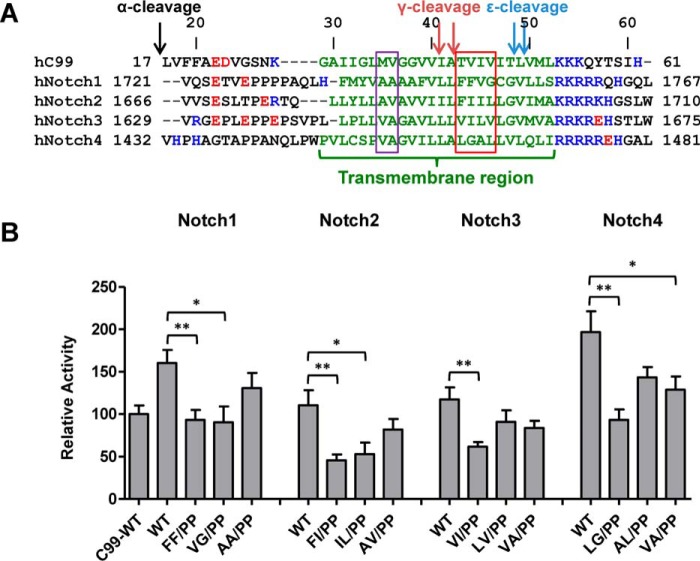
**Notch transmembrane regions homodimerize.** *A*, sequences of the TM regions of the human C99 and Notch γ-secretase substrates (C99(17–61), Notch1(1721–1767), Notch2(1666–1710), Notch3(1629–1675), Notch4(1432–1481)). Negatively and positively charged residues are *highlighted* in *red* and *blue*, respectively. Secretase cleavage sites are marked *above* the C99 sequence. The C99 TVIV-corresponding regions are marked by a *red box*, and a two-residue control region N-terminal to TVIV is marked by a *purple box. B*, Tango assays for dimerization signals of wild-type and mutant Notch family TM domains reveal that double proline mutations affect Notch TMD dimerization. *Error bars*, S.E. (*n* = 6). *, *p* < 0.05; **, *p* < 0.01; (two-tailed Student's *t* test *versus* each Notch WT).

Another important subset of γ-secretase substrates are the Notch family proteins Notch1–Notch4. Similar to C99, the C-terminal portion of the Notch TM helix is more important for cleavage by γ-secretase than other TM regions (28). Notch intracellular domain dimerization is required for Notch signaling, whereas the extracellular domain can form dimers on its own (36, 37). To test whether the TMD of Notch itself can also form dimers, we generated Tango vector constructs of 40–50-amino acid Notch TM helix-containing fragments that have been shown to be sufficient as γ-secretase substrates (28) (Fig. 11*A*). The TM fragments of all four Notch proteins elicited oligomerization signals that were as high or higher than the one for C99 (Fig. 11*B*; Tango assay reporter signals relative to that of C99), in support of the hypothesis that dimerization/oligomerization may be universal among γ-secretase substrates (20). However, in contrast to C99, introduction of double proline mutations at positions that correspond to the C99 TVIV motif (Fig. 11*A*, *red box*) did not totally abolish but significantly reduced dimerization signals (Fig. 11*B*), suggesting that local helix stability may also affect Notch TMD dimerization.

## Discussion

Aβ peptides are formed by C99 cleavage within the TM helix. Mutation of TM helix residues, especially those of the TVIV motif shown here to be important for C99 dimer formation, leads to pathological processing (38), suggesting that dimerization is an important determinant of γ-secretase recognition. Although we cannot completely exclude the possibility that the effects on dimerization and cleavage are unrelated, the lack of any clearly conserved sequence motif in the TM region of the known >90 γ-secretase substrates indicates a critical role for the local arrangement of the helices at the γ-secretase cleavage sites. Moreover, the most prominent γ-secretase substrates, including C99, Notch, and ErbB4, are able to form TM dimers. TM-mediated C99 homodimerization has been analyzed by luciferase complementation assays in cells (13), by cross-linking and co-immunoprecipitation (30), by NMR using purified or synthetic truncated C99 peptide (22–24), and by molecular modeling (17, 25), yet identification of the motif that mediates TM dimerization has remained elusive. Although G*XXX*G has been suggested as a putative C99 dimerization motif, alanine substitutions or mutations in these motifs failed to consistently alter C99 dimerization (13, 26). In this study, we used three complementary assays, Tango, BRET, and pulldown assays, to analyze C99 self-association in the context of the membrane of live cells. These assays were performed in HTL cells in which the *PS1* and *PS2* genes encoding the catalytic subunits of γ-secretase were deleted (28) to allow direct visualization of C99 interactions without γ-secretase cleavage and in the absence of any other biochemical C99 reagents or antibodies, which might themselves change the dimerization properties of target proteins. Using these assays, we validated C99 dimerization but found no evidence for higher order oligomerization in three-hybrid assays. Importantly, only mutations in four adjacent residues, Thr^43^, Val^44^, Ile^45^, and Val^46^, consistently and significantly compromised C99 dimerization. The extent of dimerization defects varied for constructs with different C termini (the BRET and two Tango assay constructs), which is consistent with a previous study (13) and implies a role of the intracellular domain in C99 topology. However, despite the quantitative differences, the qualitative effects of mutating these four residues in the contexts of three different constructs were very similar. Prior to our analysis, only two C99 residues outside of the three G*XXX*G motifs have been implicated in C99 homodimerization (31, 39), and both reside in the TVIV motif.

Even in the context of the same Tango expression construct, single Ala, single Pro, and double Ala mutation all showed similar and much smaller dimerization defects than the corresponding double Pro mutations. This indicates that both TVIV amino acid side chains and local secondary structure contribute to the stabilization of C99 in a dimer conformation. It also indicates that single Pro mutations are not sufficient to break the local secondary structure at the TVIV motif, validating the double Pro scanning approach to probe for secondary structure requirements. The TVIV motif is near the initial γ-secretase cleavage site, providing a rationale for the strong effects of disrupting its local helical structure on accessibility and preference of the initial γ-secretase cleavage site, consistent with this motif being a hot spot of FAD-linked mutations. Solid-state NMR suggests that a helix-to-coil transition near the ϵ-cut site is required for the initial γ-secretase cleavage (23), further supporting the importance of the local secondary structure in this region. Whereas C99 dimers engineered by introduced disulfide bonds showed varied effects on cleavage by γ-secretase *in vitro* (40–42), our results analyzing physiological dimerization in live cells provide strong support that disrupting C99 dimerization decreases the efficiency of γ-secretase cleavage and skews the initial cleavage site toward production of the more toxic and aggregation-prone Aβ42. Stabilizing C99 dimerization might therefore offer a potential novel therapeutic approach for controlling Aβ production.

## Experimental procedures

### DNA plasmids

The phRG-tk *Renilla* luciferase expression vector was used as transfection control. The human C99 open reading frame was synthesized by GeneWiz and subcloned into pcDNA3.0 for expression with an IgG leader sequence at the N terminus for membrane localization. For the cell-based Tango assay (27), either the coding regions of a TEV protease cleavage site (TEV site) followed by rTA or of TEV protease were cloned into the C99 expression vectors. For BRET studies, YFP and Rlu coding regions were cloned into C99 expression vectors to generate C99–YFP and C99–Rlu fusion proteins. All constructs were verified by DNA sequencing.

### Cell culture

HTL cells were a gift from G. Barnea and R. Axel (Brown University and Columbia University, respectively). They are derived from HEK293 cells with a stably integrated luciferase reporter under the control of the bacterial *tetO* operator element (43). A *PS1/PS2* knock-out HTL cell line was established previously using the CRSPR/Cas9 method (28). Cells were routinely grown in DMEM (Invitrogen) supplemented with 10% (v/v) FBS (Invitrogen) at 37 °C under humidified 5% CO_2_ atmosphere.

### Cell-based assays for C99 interactions (Tango assays)

*PS1/PS2* deletion HTL cells were split using 0.25% trypsin-EDTA into 24-well plates at a density of 50,000 cells/well. 10 ng of C99 (or its variants)–TEV site–rTA expression construct, 10 ng of C99 (or its variants)–TEV protease construct, and 5 ng of phRG-tk *Renilla* normalization luciferase expression vector were transfected together with 40 ng of pBSK plasmid control into *PS1/PS2*-deleted HTL cells the following day using 0.195 μl of X-tremeGENE 9 Reagent (Roche Applied Science) according to the standard protocol. Control Tango assays were performed with constitutively active visual arrestin (Arr(3A)) or constitutively active rhodopsin (Rho(4M)) fusion constructs as described (43). One day after transfection, cells were harvested and lysed in passive lysis buffer (Promega). Luciferase activity was measured using the Dual-Luciferase kit (Promega) according to the manufacturer's instructions. Briefly, 20 μl of cell lysates, each, were added to 96-well white Optiplates and incubated with 50 μl of LAR2 firefly luciferase substrate, followed by 50 μl of Stop & Glo® reagent to initiate *Renilla* luciferase activity. *Renilla* luciferase serves as transfection control. Relative activity was normalized against WT (set at 100%).

### Bioluminescence resonance energy transfer assay for C99 homodimerization and oligomerization (BRET assays)

*PS1/PS2* deletion HTL cells were used for transient expression of receptor constructs. Cells were plated at a density of 1.5 × 10^6^ cells/dish in sterile 10-cm tissue culture dishes. After 24 h, cells were transfected with ∼1 μg of DNA (both donor and acceptor constructs) per dish using the diethylaminoethyl (DEAE)-dextran method (44). Receptor-bearing *PS1/PS2* deletion HTL cell suspensions were used for bioluminescence and fluorescence measurements in 96-well white Optiplates, as described previously (44). Approximately 20,000 cells were studied 48 h after transfection. BRET assays were initiated by mixing 5 μm coelenterazine h (*Renilla* luciferase-specific substrate) with the cell suspension. Luminescence and fluorescence signals were collected immediately using a 2103 Envision fluorescence plate reader configured with the <700-nm mirror and with dual emission filter sets for luminescence (460 nm, bandwidth 25 nm) and fluorescence (535 nm, bandwidth 25 nm). YFP fluorescence was acquired by exciting the samples at 480 nm. Energy transfer denoted as BRET ratio was calculated based on the ratio of YFP and Rlu emission signals, as described previously (44).

Saturation BRET experiments were performed as described previously (45). Briefly, *PS1/PS2* deletion HTL cells were transfected with a fixed amount of Rlu-tagged receptor constructs as donors (0.5 μg DNA/dish) and with increasing amounts of YFP-tagged constructs as acceptors (0.15–3 μg of DNA/dish). BRET assays were performed 48 h later. The BRET ratios were plotted against the ratios of Rlu/YFP, and curves were fit and evaluated based on *R*^2^ values using GraphPad Prism version 6.0.

### γ-Secretase ϵ-cleavage assay

We described the cell-based γ-secretase ϵ-cleavage assay previously (28). Briefly, we generated fusion constructs of wild-type and mutant C99 or C79 and the transcriptional activator rTA (C99–rTA). 20 ng of C99–rTA, 5 ng of phRG-tk *Renilla*, and 40 ng of pBSK mock plasmid were co-transfected into endogenous γ-secretase containing HTL cells using X-tremeGENE 9 reagent (Roche Diagnostics) according to the manufacturer's manual. After 1 day of growth, cells were harvested and lysed for luciferase detection using the Dual-Luciferase kit (Promega). Relative activity was normalized against WT.

### Protein isolation and Western blot analysis

*PS* gene-deleted cells were transfected with the same amount of DNA as for Tango assays, using X-tremeGENE 9 reagent (Roche Diagnostics). Cells were harvested and lysed with CelLytic^TM^ M (Sigma-Aldrich) the following day. Western blot analysis was carried out using primary antibodies against FLAG tag (Sigma-Aldrich A8592), or β-actin (Abcam ab6276). The β-actin level was used as the internal control.

### Streptavidin bead pulldown assay

*PS* gene deleted HTL cells were seeded at a density of 0.8 × 10^6^/well in 6-well plates and transfected the following day with 300 ng of BirA biotin ligase encoding DNA, 500 ng of Avi–C99 encoding DNA, and 500 ng of C99–TEV site–rTA–FLAG encoding DNA with Lipofectamine® 2000 (Invitrogen) transfection reagent. After transfection, biotin solution was added to 40 μm. Cells were harvested and lysed in CelLytic^TM^ M (Sigma-Aldrich) 1 day after transfection. The crude supernatant protein extracts were incubated with prewashed Streptavidin MagBeads (GenScript) for about 30 min, followed by three washes with lysate buffer. All samples with beads were subjected to SDS-PAGE for Western blot analysis using anti-FLAG and anti-β-actin antibodies. The β-actin level was used as loading control.

### Fluorescence microscopy

Plasmids expressing C-terminal Superfolder GFP (sfGFP)-tagged WT or mutant C79 were transfected into *PS* gene-deleted HTL cells. sfGFP fluorescence was excited at 488 nm, and emission was detected at 496–518 nm 12 h after transfection. Each corresponding sfGFP view was also taken by bright field. We generated a plasmid expressing C-terminally sfGFP-tagged arrestin protein as negative control.

### Cell surface biotinylation assay

*PS* gene-deleted cells were seeded at a density of 0.8 × 10^6^/well in 6-well plates and transfected the following day with 1 μg of C99–TEV site–rTA–FLAG encoding DNA with Lipofectamine® 2000 (Invitrogen) transfection reagent. After 22 h, plates were placed on ice, medium was carefully aspirated, and cells were washed with cold PBS (20 mm potassium phosphate, pH 7.4, 150 mm NaCl). The cells were then incubated with 1 ml of PBS with 0.25 mg/ml EZlink-Sulfo-NHS-SS-biotin (Pierce) for 40 min at 4 °C. Biotinylation was stopped by the addition of 1 ml of PBS, 100 mm glycine. Cells were suspended by slow pipetting and washed once with cold TBS. Cell pellets were collected by centrifugation at 600 × *g* and lysed in CelLytic^TM^ M (Sigma-Aldrich) with 1× protease inhibitor mixture (Roche Diagnostics). Non-solubilized material was removed by centrifugation (10 min at 20,000 × *g* and 4 °C). 10 μl of supernatant plus 10 μl of 2× SDS loading buffer served as input. Remaining supernatant was incubated with prewashed Streptavidin MagBeads (GenScript) for about 30 min, followed by three washes with lysate buffer. Biotinylated proteins were eluted by reduction of the NHS-SS-biotin bond with SDS loading buffer containing 100 mm DTT for 20 min at 45 °C. Eluates were subjected to SDS-PAGE for Western blot analysis using anti-FLAG and anti-β-actin antibodies. The β-actin level was used as loading control, and FLAG-arrestin (Arr) was used as a control for a non-membrane protein.

### Site-directed mutagenesis

All site-directed mutagenesis was carried out using the QuikChange method (Agilent). All constructs were confirmed by DNA sequencing.

## Author contributions

Y. Y. and T.-H. X. designed and conducted the experiments, analyzed the data, and wrote the paper. K. G. H. and L. J. M. conducted the BRET assay. H. E. X. and K. M. designed the experiments, analyzed the data, and revised the manuscript. All authors approved the final version of the manuscript.

## Footnotes

5 Please note that the JBC is not responsible for the long-term archiving and maintenance of this site or any other third party hosted site.

## Footnotes

4 The abbreviations used are:

AD: Alzheimer's disease
Aβ: amyloid β
APP: amyloid precursor protein
Arr: arrestin
ECD: extracellular domain
AICD: APP intracellular domain
FAD: familial AD
TMD: transmembrane domain
BRET: bioluminescence resonance energy transfer
TEV: tobacco etch virus
Rlu: *Renilla* luciferase
TM: transmembrane
rTA: reverse tetracycline-controlled transactivator
YFP: yellow florescence protein.
